# Effect of oil gauze silver dressings on diabetic foot ulcers in the elderly

**DOI:** 10.12669/pjms.335.11509

**Published:** 2017

**Authors:** Chang Yan Dong, Wen Juan Liu, Rong Xiang Chi, Hong Du

**Affiliations:** 1 Chang Yan Dong, Gynecology Ward, Yantai Yuhuangding Hospital, Yantai, Shandong, 264000, China; 2 Wen Juan Liu, Gynecology Ward, Yantai Yuhuangding Hospital, Yantai, Shandong, 264000, China; 3 Rong Xiang Chi, Nursing Department, Yantai Chinese Medicine Hospital, Yantai, Shandong, 264000, China; 4 Hong Du, Cardiology Ward, Yantai Yuhuangding Hospital, Yantai, Shandong, 264000, China

**Keywords:** Diabetes, Elderly, Foot ulcer, Hydrofiber dressing, Silver

## Abstract

**Objective:**

To compare the clinical efficacy and safety of oil gauze silver dressing and silver ions dressing on diabetic ulcers in elderly outpatients.

**Methods:**

Twenty-two patients with Type-2 diabetic foot ulcers were included in the study conducted at Yantai Yuhuangding Hospital between April 2013 and April 2014. At the time of enrolment they were divided into the silver ions and oil gauze silver groups based on the order of admission. Dressings were changed twice weekly until the ulcer had healed. Clinical efficacy measures were healing outcomes and speed of healing. Adverse events were recorded.

**Results:**

The silver ions and oil gauze silver groups were comparable at baseline (P>0.05). Before treatment, the fasting blood glucose (FBG) and two hour postprandial blood glucose (2h PBG) levels were 6.88±0.50 mmol /L and 15.55±2.47 mmol/L in the oil gauze silver group, and 6.93±0.41 mmol/L and 15.23±2.58 mmol/L in the silver ions group, respectively. After treatment, the FBG and 2h PBG levels were 6.82±0.32 mmol/L and 8.67±0.86 mmol/L in the oil gauze silver group, and 6.85±0.27 mmol/L and 8.83 ± 0.61 mmol/L in the silver ions group, respectively. The healing time of foot ulcers was 23.8±2.7 days in the silver ions group and 15.8±2.5 days in the oil gauze silver group (P<0.05).

**Conclusion:**

Oil gauze silver dressings for diabetic foot ulcers were associated with favorable clinical outcomes compared with silver ion dressings, especially with respect to ulcer healing speed.

## INTRODUCTION

A diabetic foot ulcer (DFU) is a serious complication of diabetes mellitus (DM). Mishandled wound disposal in patients with DFUs is the main pathophysiologic component leading to lower extremity amputations in individuals with DM.^1^ The incidence of foot ulcers is 12%˜25% in patients with DM.^2^ Current research has confirmed that silver ions dressings yield good results in the treatment of DFU.^3^ Oil gauze silver dressings are a new type of treatment for chronic infected wound dressings, and have been widely used abroad. This study compared the effect of oil gauze silver and silver ions dressings on promoting DFU healing.

## METHODS

Twenty-two patients with DFUs who were treated at Yantai Yuhuangding Hospital between April 2013 and April 2014 were enrolled. The participants provided written informed consent. The inclusion criteria were in agreement with the 1999 World Health Organization (WHO) criteria for the diagnosis of Type-2 DM,^4^ and grade two or of the Wagner Grading System^5^ for DFUs. Patients were excluded if allergic to any component of the dressings used in the study. According to the random number table, participants were divided into the following two groups: silver ions group (n=10); and oil gauze silver group (n=12). Twenty-two patients with DFU were enrolled (age range, 61-78 years; mean age, 64.5 ± 6.7 years); the duration of diabetes was 10-23 years (mean duration, 17.2±4.3 years). The wounds were located in the plantar hallux area in 12 patients, in the plantar second metatarsal area in six patients, and in the plantar fifth metatarsal area in four cases. All of the participants had neuropathies, and the results of cultures obtained from the ulcers were positive for fungal infections. The differences were not statistically significant (P>0.05) between the two groups with respect to gender, age, duration of diabetes, DFU grades, degree of vascular lesions, and presence of injury of oral gap stealth, as shown in Table-I. The medications for diabetes control, hypertension or dyslipidemia did not change after the study.

**Table-I:** General information comparison.

*Groups*	*Gender*		*Age*	*Duration of diabetes*	*Grade of foot ulcers*		*Degree of vascular lesions*		*Presence of injury of oral gap stealth*	
	
	*Male*	*Female*			*Level 2*	*Level 3*	*Mild*	*Moderate*	*Yes*	*No*
Oil gauze silver group	9(40.9%)	3(0.5%)	64.3±7.7	17.3±4.5	9(40.9%)	3(0.5%)	9(40.9%)	3(0.5%)	3(0.5%)	9(40.9%)
Silver ion group	8(36.4%)	2(9.1%)	64.5±6.1	16.5±4.4	7(31.8%)	3(0.5%)	5(22.7%)	5(22.7%)	4(18.2%)	6(27.3%)
T value	0.043		0.118	0.121	0.047		1.255		0.491	
P value	>0.05		>0.05	>0.05	>0.05		>0.05		>0.05	

Two groups of patients were treated comprehensively, including infection control, glycemic management, and nerve nutrition. Targeted antimicrobial drugs were selected for treatment after bacterial culture results were available and drug sensitivity tests of wound secretions. At the same time, measures to reduce plantar pressure, such as the use of crutches, wheelchairs, and insoles, were designed to reduce pressure.

Normal saline was used to wash the surface of wounds, and remove cutinized hyperplastic tissues around the wound. Treatment of the wounds continued until all necrotic tissue had been removed. The wound surface was debrided and covered with sterile bandages over the silver ions dressings. The dressings were changed and medications applied to the wound twice a week. The dressings were replaced immediately when soaked until the wound was completely healed. The treatment and therapy programs of the oil gauze silver group were the same as the silver ions group. The oil silver gauze dressings were covered after debridement of the wound.

### Evaluation indicator

The wound healing time from enrollment to complete healing of the wound was recorded for both groups. “Healed completely” referred to a wound that was covered by epithelial tissue completely, with no redness and swelling, and no purulent leakage.

### Statistical analysis

All data were verified by two people before entry into the SPSS19.0 database. Statistical methods were chosen according to measurement and count data. Descriptive analysis of measurement data were reported as the mean ± 1 SD, and a t-test was applied in the intergroup comparison. Descriptive analysis of count data was reported as a frequency and percentage, and the chi-squared test was applied in the intergroup comparison. A P <0.05 difference was statistically significant.

### Ethic Approval

This study was approved by the Ethic committee of The Yan Tai Yuhuangding Hospital. [No.2013092]

## RESULTS

### Blood glucose value comparison

There were no statistically significant differences between the two groups of patients with respect to FBG and 2h PBG levels before and after treatment (P>0.05; Table-II).

**Table-II:** Blood glucose value comparison.

*Groups*	*FBG*		*2hPBG*	

	*Before treatment*	*After treatment*	*Before treatment*	*After treatment*
Oil gauze silver group	6.88±0.50	6.82±0.32	15.55±2.47	8.67±0.86
Silver ion group	6.93±0.41	6.85±0.27	15.23±2.58	8.83±0.61
T value	0.206	0.289	-0.321	0.667
P value	0.834	0.775	0.752	0.513

### Foot ulcer healing time comparison

The DFU healing time was 15.6±2.4 d and 23.7±2.6 d in the oil gauze silver and silver ions groups, respectively P<0.05; Table-III.

**Table-III:** Foot ulcer healing time comparison.

*Groups*	*n*	*healing time*	*t value*	*P value*
Oil gauze silver group	12	15.8±2.5	8.281	0.000
Silver ion group	10	23.8±2.7		

## DISCUSSION

The severity of DFU and the level of infection were positively correlated. When the bacterial population overloads a DFU, the bacteria compete for oxygen and nutrients with normal cells. At the same time, the bacterial endotoxins and proteases, such as bacterial products, hinder wound healing at each stage, thus causing wound healing difficulties. Infection control to reduce the bacterial load reduces oxygen transport and utilization in wounds, and the chronic inflammatory reaction, thus shortening the healing time of DFUs.

Silver ions have a strong bactericidal and bacteriostatic effect, and can block the microbial respiratory chain, damage the microbial cell membrane, and combine microbial RNA and DNA to inhibit execution of normal replication, transcription, and translation, thus producing a powerful killing effect on various microorganisms, including bacteria, viruses, fungi, and protozoa under aerobic and anaerobic conditions.^6^ Silver ions, which cannot distinguish between normal and diseased tissues at the site of wounds, are toxic to keratinocytes and myofibroblasts, which can promote normal tissue growth. Therefore, the silver ions kill the keratinocytes and myofibroblasts when killing bacteria and viruses, such as microorganisms. The low release rate of silver ions has a less antibacterial effect. Therefore, there is a need to adjust the balance between antibacterial activity and cytotoxicity of silver ions to promote DFU healing.

This study showed that the average healing time of plantar ulcers in the oil gauze silver group was shorter than the silver ions group, and the difference was statistically significant (P<0.05). The study conducted by Ziegler^7^, which included 86 patients with foot ulcers, showed that oil silver gauze dressings had lower cytotoxicity and a silver ion release rate than two other common silver ion dressings, which was consistent with the results of this study. Oil silver gauze dressings are new dressings and are a combination of rough waterproof polyester fibers and efficient antimicrobial silver ions. The silver ions are stored in the polyester fibers in the compound form. When the polyester fibers are exposed to wound exudates, the silver ions are released immediately, specifically bind to the bacteria, and effectively kill the bacteria. The low release rate of silver ions ensures a long duration of an effective concentration of silver ions, and a long duration of bactericidal action. Studies have shown that there are fewer silver ions and less toxicity of oil silver gauze dressing than other dressings surrounding wound tissues.^8^-^10^ Polyester fibers are mainly composed of a triglyceride-based hydrophilic cream, on which there are many pores with a 1-mm diameter, thus allowing exudate, dead bacteria, and endotoxin to reach the second dressing. In addition, polyester fibers can prevent granulation tissue from sticking firmly to dressings, reducing pain and trauma during dressing removal and improving comfort. Neutral triglycerides further enhance the non-adherent properties, softening tissues around the wound, thereby preventing shrinkage and reducing scarring.

In short, the oil silver gauze dressings have properties that differ from other silver dressings. In the wound environment, oil silver gauze dressings do not produce stimulation to normal granulation tissue. Indeed, the role of oil silver gauze dressings if focused on the infected tissue, exerting anti-inflammatory effects. The resulting shortened healing time of oil silver gauze dressings compared with silver ions dressings warrants further validation and promotion in DFU care applications.

### Study Limitation

The small sample size is a limitation. Because ethically one cannot compromise the health of patients by withholding the appropriate treatment, larger sample size was not available. In future the study will include more patients in a reasonable sample size to confirm these observations.

### Authors’ Contribution

**CYD** did clinical and experimental studies, data acquisition and analysis, statistical analysis.

**WJL** did definition of intellectual content, literature research.

**RXC** did manuscript editing and review.

**HD** is responsible for guarantor of integrity of the entire study, study concepts, study design.
